# Effect of the Addition of Thermoplastic Resin and Composite on Mechanical and Thermal Properties of Epoxy Resin

**DOI:** 10.3390/polym14061087

**Published:** 2022-03-08

**Authors:** Jingyu Wu, Chenggao Li, Bahetihan Hailatihan, Longlong Mi, Yeerna Baheti, Yuze Yan

**Affiliations:** 1 College of Civil Engineering, Shandong Jianzhu University, Jinan 250101, China; wujingyu1985@163.com; 2 Key Laboratory of Smart Prevention and Mitigation of Civil Engineering Disasters of the Ministry of Industry and Information Technology, Harbin Institute of Technology, Harbin 150090, China; clementinematin1734@gmail.com (B.H.); mll3312967802@163.com (L.M.); y1973527128@163.com (Y.B.); yyz15126030594@163.com (Y.Y.); 3 Key Laboratory of Structures Dynamic Behavior and Control, Ministry of Education, Harbin Institute of Technology, Harbin 150090, China; 4 School of Civil Engineering, Harbin Institute of Technology, Harbin 150090, China

**Keywords:** epoxy resin, thermoplastic resins and composites, mechanical properties, thermal properties, improvement mechanism, application prospect analysis

## Abstract

When the thermoplastic composites reach the service limits during the service, the recovery and utilization are the key concerns. Meanwhile, the improvement of strength, toughness and durability of epoxy resin is the effective method to prolong the service life of materials and structures. In the present paper, three kinds of thermoplastic resins (polypropylene-PP, polyamide 6-PA6 and polyether-ether-ketone-PEEK) and composites (carbon fiber-PEEK, glass fiber-PA6 and glass fiber-PP) were adopted as the fillers to reinforce and toughen the epoxy resin (Ts). The mechanical, thermal and microscopic analysis were conducted to reveal the performance improvement mechanism of Ts. It can be found that adding thermoplastic resin and composite fillers at the low mass ratio of 0.5~1.0% brought about the maximum improvement of tensile strength (7~15%), flexural strength (7~15%) and shear strength (20~30%) of Ts resin. The improvement mechanism was because the addition of thermoplastic fillers can prolong the cracking path and delay the failure process through the load bearing of fiber, energy absorption of thermoplastic resin and superior interface bonding. In addition, the thermoplastic composite had better enhancement effect on the mechanical/thermal properties of Ts resin compared to thermoplastic resin. When the mass ratio was increased to 2.0~3.0%, the agglomeration and stress concentration of thermoplastic filler in Ts resin appeared, leading to the decrease of mechanical and thermal properties. The optimal addition ratios of thermoplastic resin were 0.5~1.0% (PEEK), 1.0~2.0% (PA6) and 0.5~1.0% (PP) to obtain the desirable property improvement. In contrast, the optimal mass ratios of three kinds of composite were determined to be 0.5~1.0%. Application prospect analysis indicated adding the thermoplastic resin and composite fillers to Ts resin can promote the recycling and reutilization of thermoplastic composites and improve the performance of Ts resin, which can be used as the resin matrix, interface adhesive and anti-corrosion coating.

## 1. Introduction

Polymers has been widely used in aerospace, automation, civil engineering, marine engineering and other fields [1,2,3,4,5]. According to the composition and type, it mainly includes the thermosetting resin (such as epoxy) with the three-dimensional network crosslinking structure and thermoplastic resin with the linear molecular structure. For epoxy resin, it has excellent mechanical properties, convenient molding process and better bonding strength with the other medium, which can be used in the resin matrix, interface adhesive and coating for the repairing and reinforcing of engineering structures [6,7,8]. However, the three-dimensional network structure of epoxy resin leads to the poor fracture toughness, which is easy to bring about the cracks and concentration under the complex load conditions, resulting in fatigue damage and ultimate failure [9,10,11]. At the same time, the hydroxyl groups in the molecular structure are easy to react with water molecules, which leads to the durability degradation, such as the hydrolysis and plasticization [12,13,14,15]. Compared with thermosetting resin, thermoplastic resin has excellent fracture toughness, moisture, thermal and fatigue resistances [16,17]. In addition, it can be recycled and does not pollute the environment, which has gradually achieved great application potential in structural engineering application [18]. To sum up, the mechanical/interfacial reinforcing, toughening and durability improvement of thermosetting epoxy resin [19,20] through adding some nanoparticles, such as carbon nanotubes, graphene and thermoplastic particles, and the recycling of thermoplastic resin are two main problems to be solved.

An effective method to reinforce the epoxy resin can be adopted through the continuous fiber to prepare fiber reinforced epoxy composites, such as carbon fiber, glass fiber, basalt fiber reinforced epoxy composites [21,22,23,24]. Since the fibers have high tensile strength, adding them to the epoxy can achieve the high-performance composites. On the other hand, the mechanical properties and durability of epoxy resin can be further improved by adding some functional nanoparticles to epoxy resin. Tian et al. [25] studied experimentally the effects of titania content and sample preparation methods on the mechanical performances of epoxy. They found that the tensile strength of epoxy increased by up to 30% compared with the control for the optimal mixture ratio (4%) of titania. The improvement mechanism was attributed to the formation of super-monomer through the uniform dispersion technique, which can also reduce the size of agglomerations of more than 5 microns. Uthaman et al. [26] investigated the thermal, mechanical, and water uptake properties of epoxy through adding the surfactant modified multi-walled carbon nanotubes. The homogenous distribution of the carbon nanotubes in the epoxy can be achieved, which contributed to the obvious increase in the mechanical and thermal properties. The increase mechanism was because the free volume between the polymer chains was reduced and the chain segmental mobility was restricted, which brought about the strong interfacial bonding and an efficient load transfer capability between the carbon nanotubes and epoxy matrix. Additionally, the carbon nanotubes reduced the surface wettability of epoxy, leading to the increase of contact angle and the reduction of water uptake. Baghdadi et al. [27] investigated the effects of functional iron oxide including the polydopamine and trimethoxysilane on thermal and mechanical properties of epoxy resin. It can be found that the maximum improvements in tensile strength (~34%) and fracture toughness (~13%) were obtained when adding the pristine iron oxide. Carbas et al. [28] analyzed the effects of carbon black nanoparticles on the stiffness, strength and deformation of epoxy adhesive. Thy found that the strength and stiffness of epoxy decreased with the increase of the carbon black amount. By contrast, an increase of the deformation was observed with the increase of carbon black concentration, indicating the ductile fracture behavior. Haeri et al. [29] prepared the silica-functionalized graphene oxide nanosheets and analyzed the its enhancement effect on the mechanical properties of epoxy. They observed that when 1 wt.% of the silica modified nanosheets was added to the epoxy, the tensile strength, storage modulus, cross-linking density and glass transition temperature (Tg) of epoxy were remarkably increased. The improvement mechanism was because the nanosheets could enhance the resistance against crack propagation through creating many stress concentrating areas, dissipating the stress and preventing the stress transfer to the epoxy. To sum up, the addition of nanoparticles can improve the mechanical properties and durability of epoxy resin. However, due to the complex preparation process and high price of above nanoparticles, their applications in the reinforcement and toughening of epoxy resin may be limited to popularize.

The common thermoplastic resins in aerospace, construction engineering applications mainly include polyethylene resin (PE), polypropylene resin (PP), polyamide resin (PA), polycarbonate resin (PC) and polyether ether ketone resin (PEEK) [30,31,32,33,34,35]. For the above thermoplastic resins, their molecular weight structures are generally linear or branched, and there is no chemical bond formed in the molecular chain, which determines that they can be recycled and reused through the process of heating-melting and cooling-molding. Okan et al. [36] summarized the recycling of thermoplastic resin was associated with many difficulties, such as separation, sorting and cleaning operations, instability of selective garbage separation programs, high transport and electricity costs, etc. Recycling methods were also diverse, mainly including the refurbishing, mechanically reshaping, chemically treating, thermally utilizing, etc. Recently, some novel process technologies have emerged, such as application in carbon capture or synthesis of carbon nanostructures from the thermoplastic wastes. Exconde et al. [37] focused on the materials selection of virgin polymer and recycled post-consumer thermoplastic resin used for 3D printer filaments. They found that the virgin low-density polyethylene and recycled polyethylene terephthalate were the optimal materials as an alternative filament. Kim et al. [38] prepared the unidirectional carbon-fiber/thermoplastic/epoxy prepreg through embedding the microcapsules containing a healing agent for a thermoplastic-toughened epoxy matrix. It can be found that the intact microcapsules filled with healing agent had significant potential to decrease some small-scale damages in the composite. Sun et al. [39] investigated the effects of polysulfone on the mechanical and thermal properties of epoxy resin through the mechanical tests and microstructure analysis. They found that polysulfone resin had good compatibility with epoxy resin, which can promote the curing reaction of the epoxy resin. Furthermore, the fracture toughness and impact strength were significantly improved owing to the bicontinuous phase structure of polysulfone/epoxy blends. Dzul-Cervantes et al. [40] studied the effect of moisture absorption on the fiber-matrix interfacial shear strength between a polysulfone-modified epoxy matrix and silane surface-treated carbon fiber. It can be observed that both the silane treatment and the polysulfone modification of the epoxy resin can enhance the composite’s resistance to moisture attack. So far, the combined research on the reinforcing, toughening and durability improvement of thermosetting epoxy and the secondary utilization of thermoplastic resin were still less. Furthermore, the effect mechanism of the addition of thermoplastic resin and its composites on mechanical and thermal properties of epoxy resin were not clear.

According to the above analysis, it is key to improve the mechanical/interfacial reinforcement and durability improvement of epoxy resin. At the same time, the secondary utilization of thermoplastic resin plays a significant role of the recycling of engineering materials. Therefore, it was necessary to study the improvement mechanism of thermoplastic resin and its composite on the mechanical and thermal properties of epoxy resin. In the present study, three kinds of thermoplastic resins (polypropylene-PP, polyamide 6-PA6 and polyether-ether-ketone- PEEK) and composites (carbon fiber-PEEK, glass fiber-PA6 and glass fiber-PP) were adopted as the fillers to reinforce and toughen the epoxy resin (Ts). The mechanical, thermal and microscopic analysis were conducted to reveal the performance improvement mechanism. The design of optimal thermoplastic filler was proposed based on the performance improvement. Finally, the application prospect of adding thermoplastic resin and composite fillers to Ts resin was analyzed to provide a new idea for the polymer application. The main object of present work is to improve the performances of epoxy and the recycling application of thermoplastic resin, which promote the development of material, environment and economy.

## 2. Materials and Methods

### 2.1. Raw Materials

In the present paper, the bisphenol A epoxy resin (marked as Ts) from Dagong Composite Company (Linyi, Shandong, China) and amine curing agents were adopted. The mass ratio of the above two components was 1:0.345. Three thermoplastic resins and composites were used as the fillers as following. The polypropylene (PP), polyamide 6 (PA6) and polyether-ether-ketone (PEEK) were adopted as the thermoplastic resin filler, and carbon fiber reinforced polyether-ether-ketone (CF/PEEK) and glass fiber reinforced polypropylene (GF/PP) and polyamide 6 (GF/PA6) prepreg tape were adopted as the thermoplastic composite fillers. For GF/PP, it was produced by CIMC Composite Technology Co., LTD. (Qingdao, China). The melting temperature of polypropylene was about 164~170 °C, and the thickness of prepreg tape was 0.3 mm. The density was 1.51 g/cm^3^, and fiber mass fraction was about 57.5%. Nominal tensile strength was 524 MPa (±29.5 MPa) along the fiber direction and 5.6 MPa (±0.35 MPa) perpendicular to the fiber direction. For CF/PEEK, the carbon fiber (12 k, Shanghai Petrochemical, Shanghai, China) was adopted and the polyether-ether-ketone (PEEK-330G) was bought from Zhongyan Polymer Materials Co., LTD (Changchun, Jilin, China). The density of prepreg tape was 1.3~1.4 g/cm^3^, and the fiber mass fraction was 55~60%. The tensile strength and tensile modulus were 1400~1700 MPa and 130~170 GPa along the fiber direction, respectively. It should be noted that the thermoplastic composite fillers with different fiber composition were used to simulate the recycling and reuse of carbon fiber and glass fiber in different application fields. At the same time, the above different thermoplastic fillers are added to resin to obtain the epoxy products with the different mechanical and thermal properties. Through the comparison and optimization, the epoxy resin with better performance will be achieved. In addition, the thermoplastic resin fillers with different nature and properties were used to select the optimal filler to achieve the maximum improvement of mechanical and thermal properties of epoxy resin.

### 2.2. Sample Preparation

The test conditions of adding thermoplastic resin and its composites to Ts resin were shown in Table 1. It can be seen that the addition mass ratio of three kinds of thermoplastic resins and composites were 0.5%, 1%, 2% and 3%, respectively. The preparation process of Ts epoxy sheet with and without the fillers was as follows. Firstly, the thermoplastic resins and composites prepreg tape were broken into powder and fine particles with the fiber length of 2~3 mm using the grinding machine. Then the prepared fillers were evenly dispersed into Ts resin using the homogenization machine (ZYMC-350VS) with the speed of 3000 r/min for 200 s. Finally, the curing agents was added into the epoxy and then evenly stirred for 10 min, followed by oscillated with ultrasonic wave for 15 min to remove the bubbles. After the above process, Ts resin with the fillers were poured into a silicone rubber mold and cured at room temperature for 24 h and 60 °C for 24 h in the oven. After the curing, the resin sheets were cut into different sizes and polished with a polishing machine to conduct the mechanical, thermal and microstructure tests. The mechanical tests included the tensile, flexural and in-plane shear tests, and the thermal tests included the dynamic mechanical analysis and thermogravimetric analysis, as shown in Table 1.

### 2.3. Mechanical Tests

According to ASTM D 638-2010, the tensile tests of Ts and Ts/filler sheets were conducted through using the universal tensile machine (DHY-10080, Shanghai, China). The samples were prepared with the dumbbell shape as specified in the above standard. The extensometer was used to monitor the deformation during the tensile process. The crosshead displacement rate of tensile machine grip was 2 mm/min. It should be noted that the lower crosshead displacement rate compared to the standard was to observe the fracture behavior during the tensile process. Three samples were prepared and tested to obtain the average. Furthermore, the tensile stress-strain curves were also obtained to analyze the tensile behavior.

Based on the ASTM D7264-07, the flexural tests of Ts and Ts/filler sheets were conducted through the same universal tensile machine. The sample thickness was cut into 2 mm, and the span-to thickness ratios was 16:1. According to the above selection, the rectangular size of the sample was determined to be 40 mm × 10 mm × 2 mm. The displacement rate of tensile machine grip was set to 2 mm/min. Five samples were repeated to obtain the average flexural strength.

The shear device was designed in Harbin Institute of Technology [41] to conduct the in-plane shear tests of Ts and Ts/filler sheets. The samples were prepared with the rectangle size of 10 mm (height) × 10 mm (width) × 2 mm (thickness). Five samples were repeated for each condition to obtain the average shear strength. The crosshead displacement rate of universal tensile machine (DHY-10080, Shanghai, China) was set to 1.0 mm/min.

### 2.4. Thermal Properties Tests

The thermal properties of Ts and Ts/filler were characterized through the dynamic mechanical analysis (DMA-Q800, TA Instrument, New Castle, DE, USA). The samples were cut into the rectangle size of 40 mm (length) × 5 mm (width) × 2 mm (thickness). The clamp type adopted the single cantilever beam, and the loading frequency was 1 Hz and the heating rate was 5 °C/min from room temperature to 200 °C. Two samples were tested to obtain the average glass transition temperature (Tg). In addition, the storage modulus was obtained to analyze the effect of fillers on the stiffness of Ts resin.

The mass variations of Ts and Ts/filler from room temperature to 800 °C were obtained through the thermogravimetric analysis (TGA, NETZSCH STA 449C, Hessen, Germany). The sample was prepared into powder around 10 mg and 20 mL/min dry air flow was applied during the test. The mass decomposition rate at elevated temperatures of Ts resin was further analyzed.

### 2.5. Fourier Infrared Spectroscopy Test

Fourier infrared spectra of Ts resin before and after adding the fillers were analyzed through FTIR783 (Perkin-Elmer spectrometer, Waltham, MA, USA). The samples were cut and ground into powder about 2 mg mixed with 200 mg of KBr. Each spectrum was obtained by the scanning of 64 times in the wave number range of 400–4000 cm^−1^ with a resolution of 4 cm^−1^. The variations of functional groups were analyzed to evaluate the effect of fillers on the Ts resin.

### 2.6. Scanning Electron Microscopy (SEM)

The surface morphology of Ts and Ts/filler were observed through the scanning electron microscope (SEM, VEGA3). Before the tests, the samples were vacuumed and sprayed with gold about 180 s to increase the electrical conductivity. The test frequency, electric current and voltage amplitude were set as 1000 Hz, 0.7 A and 30 kV, respectively. The samples after the tensile tests were adopted to analyze the fracture failure mode.

## 3. Results and Discussion

### 3.1. Mechanical Properties

#### 3.1.1. Tensile Properties

**Figure 1:**
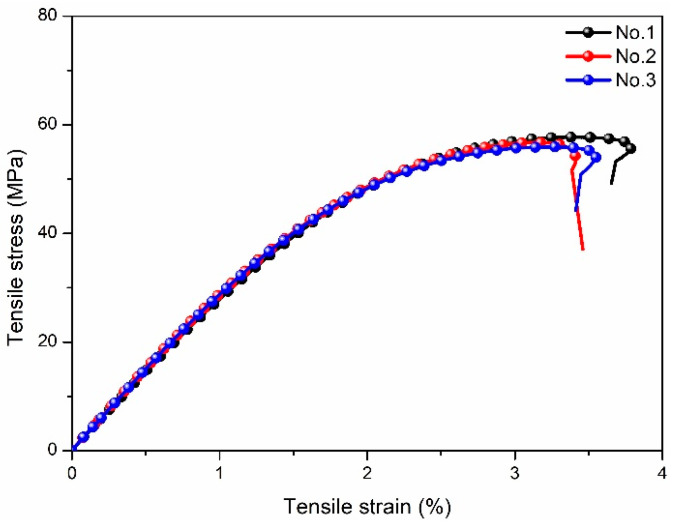
Tensile stress-strain curves of Ts resin.

Before the thermoplastic fillers were added, the tensile properties of Ts resin were obtained and shown in Figure 1 (stress-strain curves). As shown, the tensile stress-strain curves showed a two-stage variation trend, including the initial elastic deformation stage (linear and nonlinear elasticities) and the subsequent plastic deformation stage. According to the slope of linear elastic stress-strain curve, the tensile modulus of Ts resin was determined to be 2.86 GPa. In the plastic deformation stage, the tensile strength and elongation at break of Ts resin were obtained to be 56.81 MPa and 3.26%, respectively. It can be seen that Ts resin had good toughness and plastic deformation ability. In addition, it can be found that the tensile stress-strain curves of three samples were similar, which indicated the better stability of experimental data.

**Figure 2:**
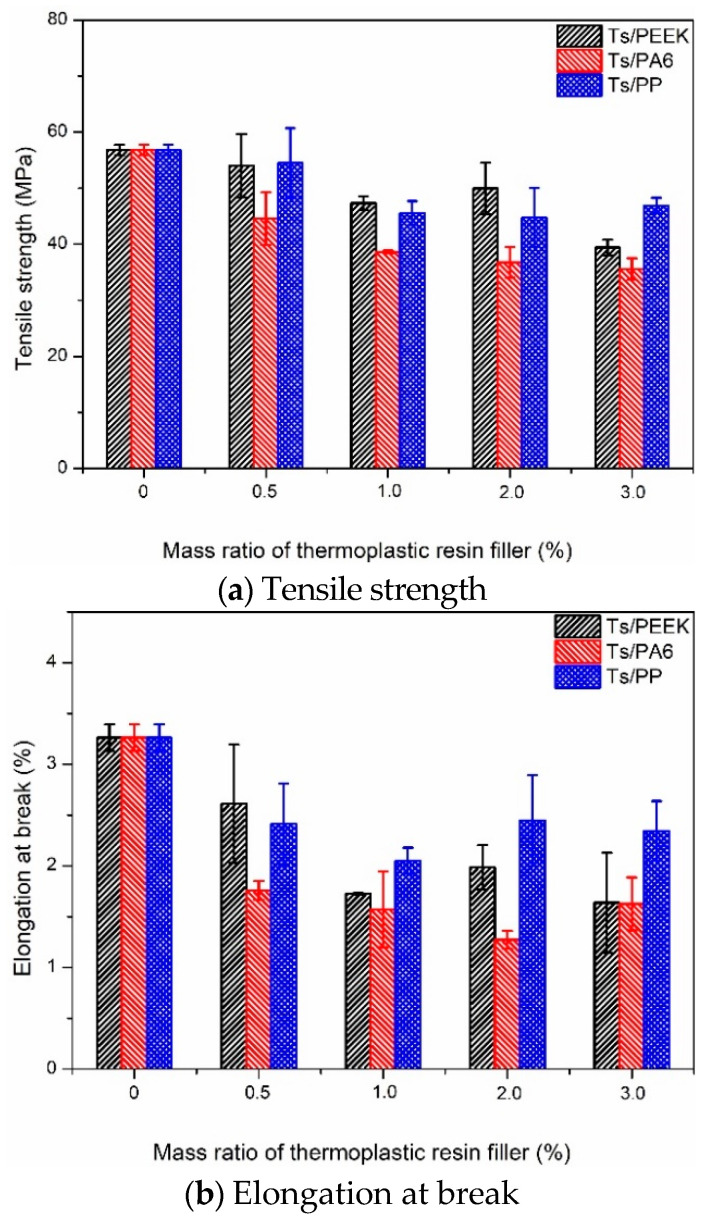
Effects of thermoplastic resin fillers on the tensile properties of Ts resin of (**a**) tensile strength and (**b**) elongation at break.

After obtaining the tensile properties, the effects of thermoplastic resin filler on the tensile properties of Ts resin were analyzed and shown in Figure 2. As shown in Figure 2a, it can be found that the addition of three kinds of thermoplastic resin fillers led to the decrease of tensile strength for Ts resin. Furthermore, the strength decrease proportion increased with the increase of mass fraction of filler from 0.5 to 3.0%. The above decrease may be due to the interface incompatibility between Ts resin and thermoplastic resin, which was further aggravated with the increase of filler mass fraction. Incompatible interfaces were more likely to lead to results in weaker load transfer capability and crack initiation and propagation in the tensile process, leading to the decrease of tensile strength and the increase of mean differences with a larger standard deviation [42]. Figure 2b shows the elongation at break had the obvious decrease owing to the addition of thermoplastic resin fillers, and the decrease mechanism was similar to the tensile strength. In addition, it can be found that the tensile properties of Ts/filler composites degraded less for the addition ratio of 0.5%, especially for the fillers of PEEK and PP. With the increase of the addition ratio, the thermoplastic resin fillers were easy to agglomerate in Ts resin, resulting in the stress concentration and the decrease of tensile strength and elongation at break.

**Figure 3:**
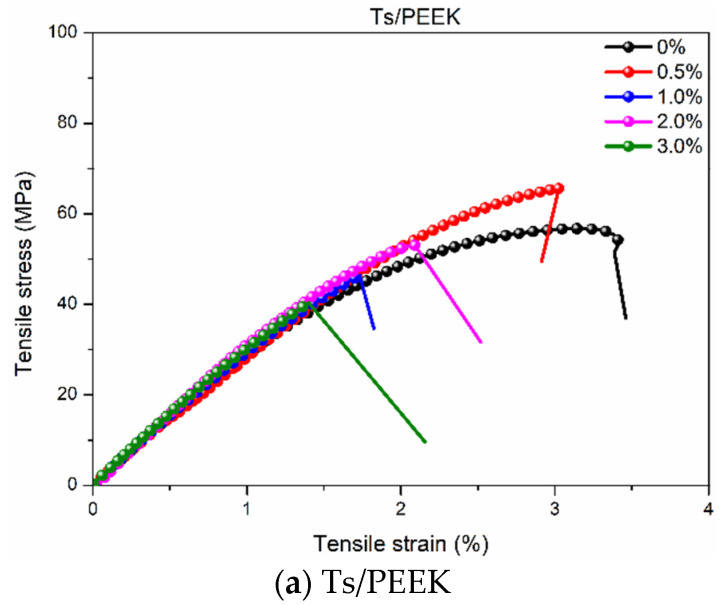

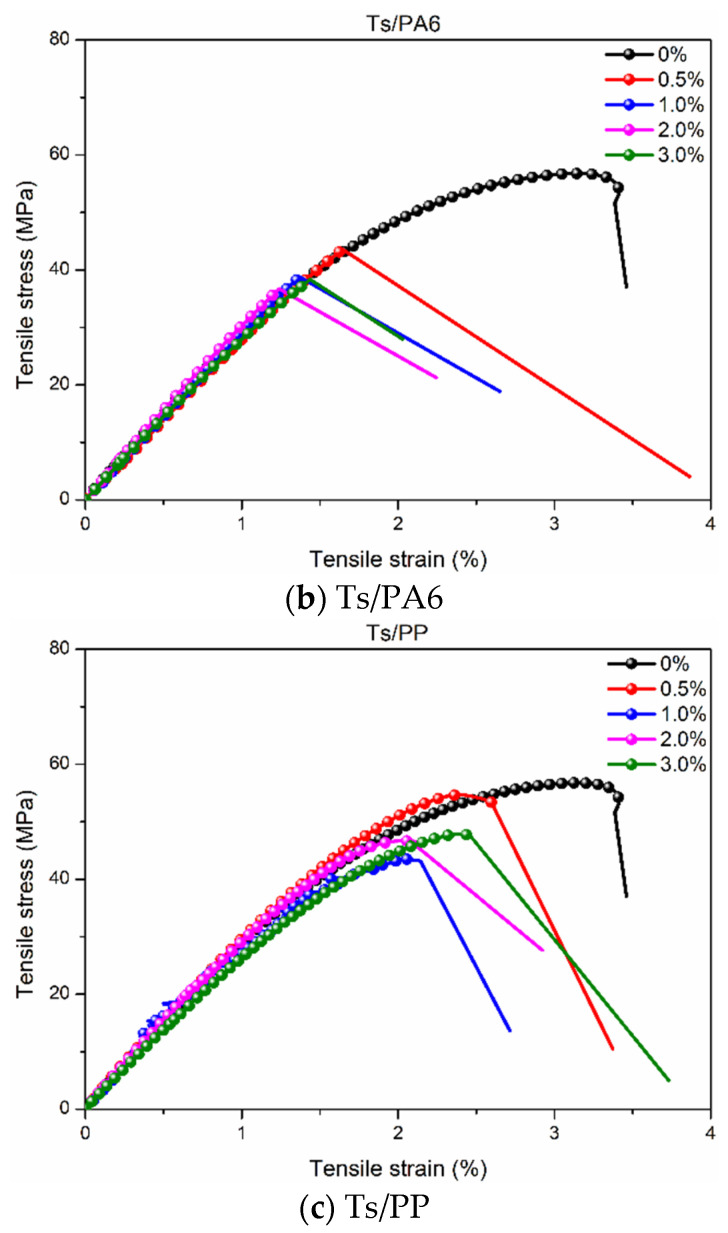
Effects of thermoplastic resin fillers on the tensile stress-strain curves of Ts resin of (**a**) Ts/PEEK, (**b**) Ts/PA6 and (**c**) Ts/PP.

Figure 3 shows the effects of thermoplastic resin fillers on the tensile stress-strain curves of Ts resin. Similar to the tensile strength and elongation at break, adding the thermoplastic resin fillers led to the weakening and embrittlement of the composite owing to the interface incompatibility and discontinuity between Ts resin and thermoplastic resin. Furthermore, the plastic deformation stage of the tensile stress-strain curve was weakened. In addition, it can be found that the above “weakening effect” was further aggravated with the increase of the content of thermoplastic resin filler. Therefore, how to add the interfacial bonding between thermoplastic resin and Ts resin was the key to improve its tensile properties.

**Figure 4:**
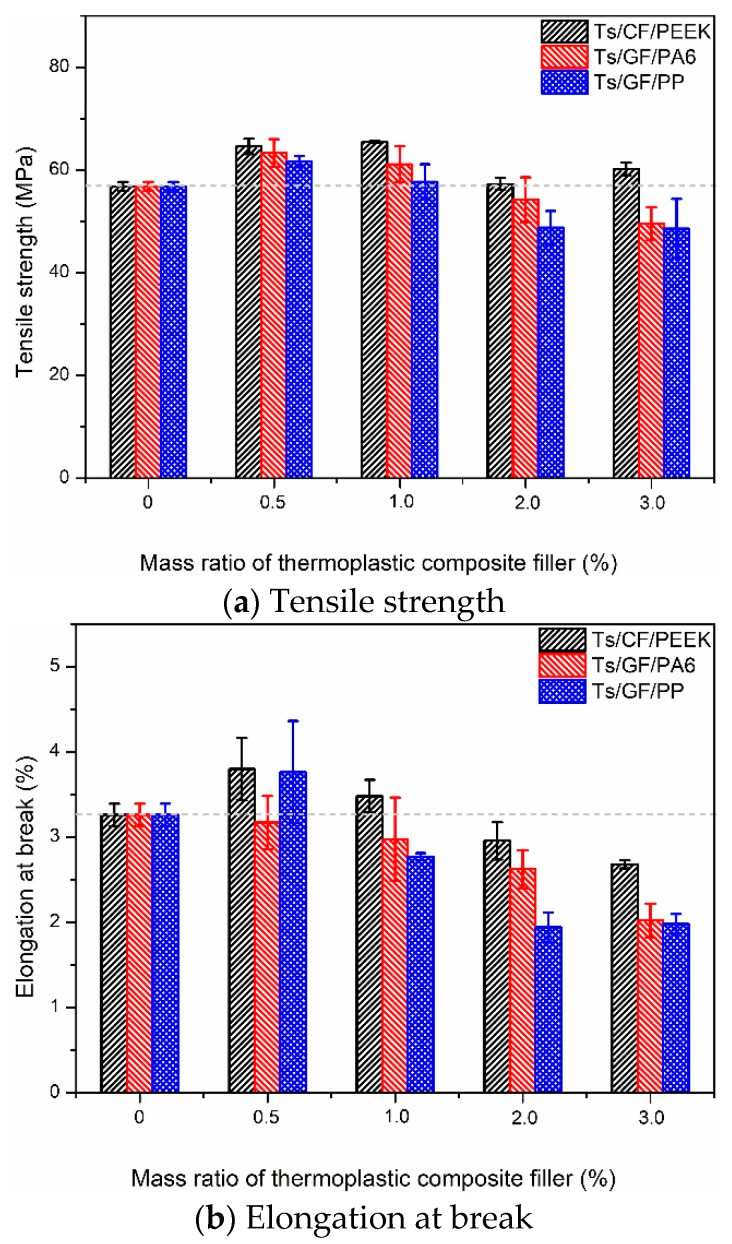
Effects of thermoplastic composite fillers on the tensile properties of Ts resin of (**a**) tensile strength and (**b**) elongation at break.

**Figure 5:**
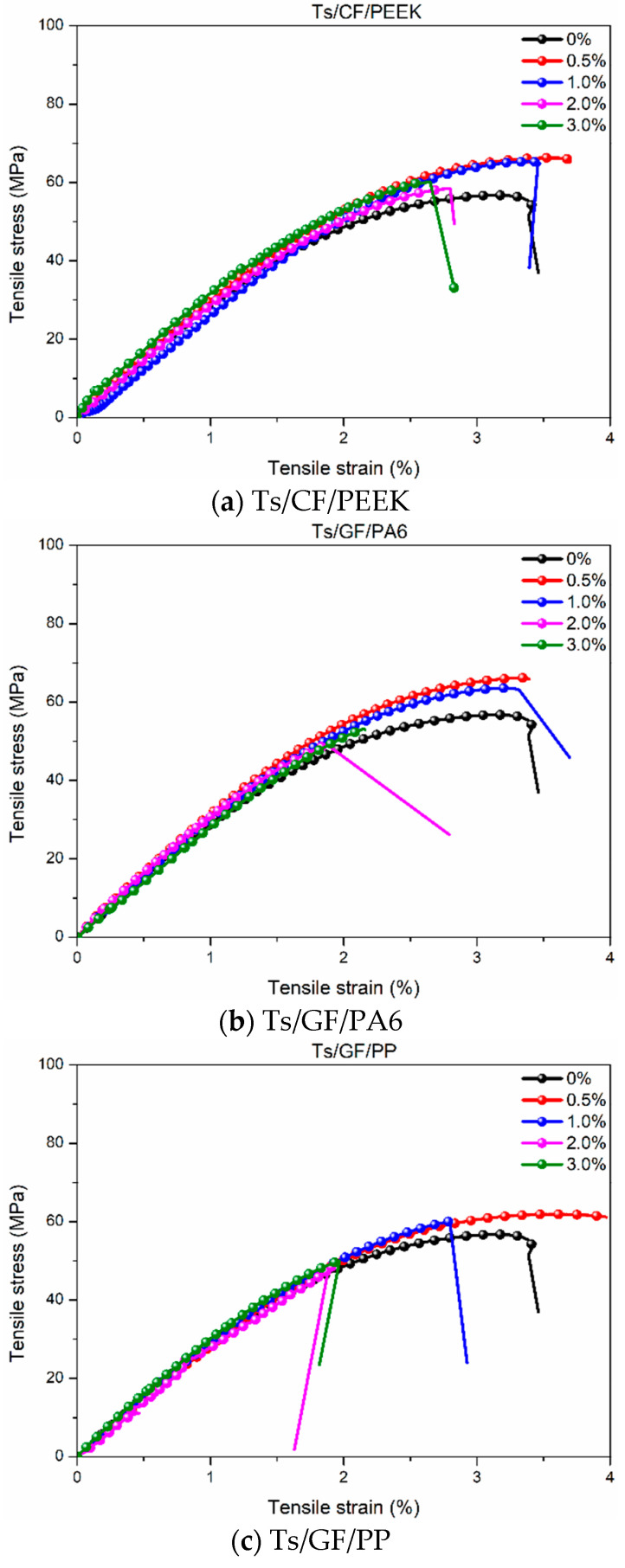
Effects of thermoplastic composite fillers on the tensile stress-strain curves of Ts resin of (**a**) Ts/CF/PEEK, (**b**) Ts/GF/PA6 and (**c**) Ts/GF/PP.

After analyzing the effect of thermoplastic resin filler on the tensile properties of Ts resin, the effects of thermoplastic composite fillers on the tensile properties were shown in Figure 4 and Figure 5. As shown in Figure 4, it can be found that the addition of thermoplastic composite filler can significantly improve the tensile strength and elongation at break of Ts resin when the filler mass ratio was 0.5~1.0%, similar to the effect of polyethersulfone-modified on epoxy [43]. This was because carbon and glass fibers as the connecting medium can effectively bond the thermoplastic resin and Ts resin owing to the intermolecular chemistry and mechanical interactions. Furthermore, the interface incompatibility and discontinuity between Ts resin and thermoplastic resin were further compensated to obtain the ability to resist cracking, leading to the increase of tensile strength (Figure 4a). Meanwhile, the added thermoplastic fillers may form an interpenetrating network with the epoxy, which increased a constraining closure effect on crack propagation and further expansion [44]. In addition, the higher plastic deformation ability of thermoplastic resin (PEEK, PA6 and PP) was also fully utilized, which led to the significant increase of the elongation at break of Ts resin (Figure 4b). When the content of thermoplastic composite filler was more than 1%, the tensile strength and elongation at break of Ts resin decreased on the contrary. This was due to more filler content formed the agglomeration effect in Ts resin, leading to the stress concentration and the decrease of tensile strength and elongation at break [26,45].

Figure 5 shows the effects of thermoplastic composite fillers on the tensile stress-strain curves of Ts resin. It is worth mentioning that stress-strain curves were used to analyze elastic modulus, fracture failure, toughness and ultimate elongation at break. Similar to the tensile strength and elongation at break, adding the thermoplastic composite fillers brought about the higher toughness and strength, leading to the right and upper shifts of stress-strain curves for the filling content of 0.5~1.0%. This toughness improvement might be attributed to the excellent interfacial bonding can absorb the crack energy [46]. Furthermore, the crack could not avoid progressing through the tough thermoplastic composite fillers phase once composite formed a continuous phase. With the increase of filler content, the tensile stress-strain curves gradually became weakened and brittle owing to the agglomeration effect and discontinuous phase [47]. In addition, it can be found that the addition of CF/PEEK brought about the maximum improvement of toughness and strength of Ts resin compared to GF/PA6 and GF/PP.

The increase percentage of tensile properties for Ts resin after adding the thermoplastic fillers was obtained. It can be seen that the addition of thermoplastic resin filler did not improve the tensile strength and elongation at break of Ts resin. In comparison, the thermoplastic composite filler brought about the maximum increase percentage of 15.21% (CF/PEEK), 11.45% (GF/PA6) and 8.28% (GF/PP) for the tensile strength, 16.49% (CF/PEEK) and 15.44% (GF/PP) for the elongation at break. Overall, the mass ratio of thermoplastic composite filler should be controlled at 0.5~1%, which was helpful to improve the tensile properties of Ts resin. When the mass ratio increased to 2.0~3.0%, Only the addition of CF/PEEK showed a positive effect of tensile strength for Ts resin.

#### 3.1.2. Flexural Properties

**Figure 6:**
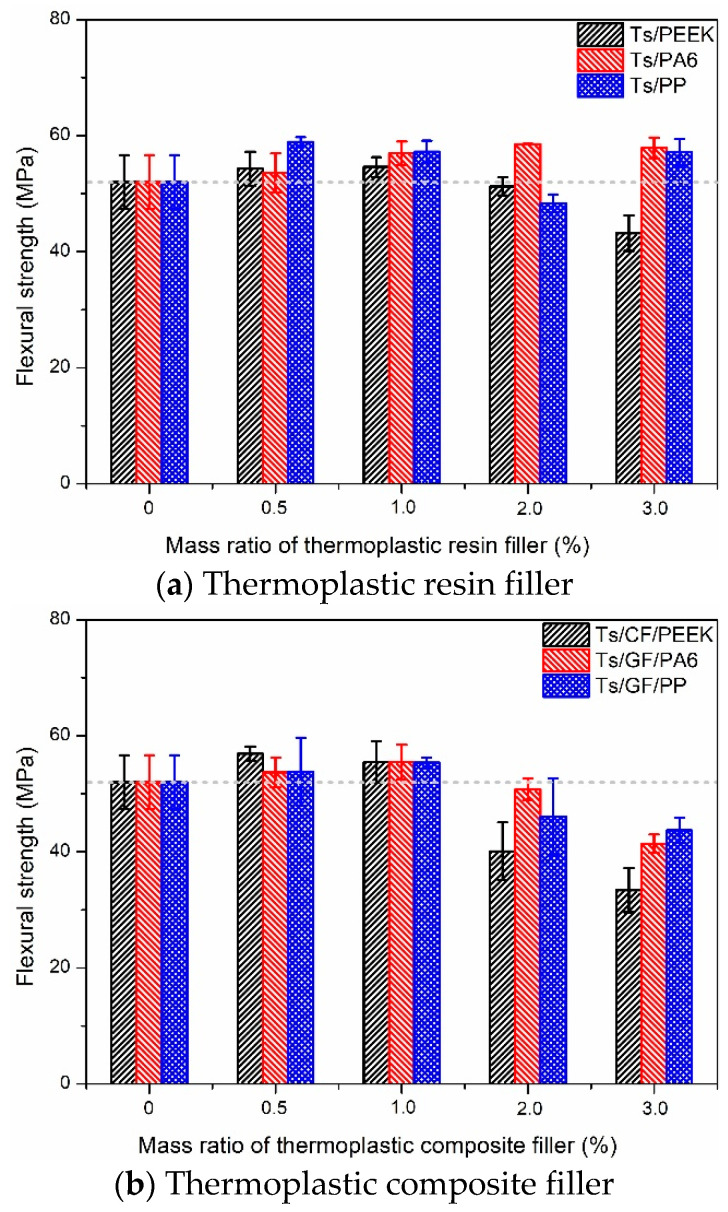
Effects of thermoplastic resin and composite fillers on the flexural strength of Ts resin of (**a**) thermoplastic resin filler and (**b**) thermoplastic composite filler.

**Figure 7:**
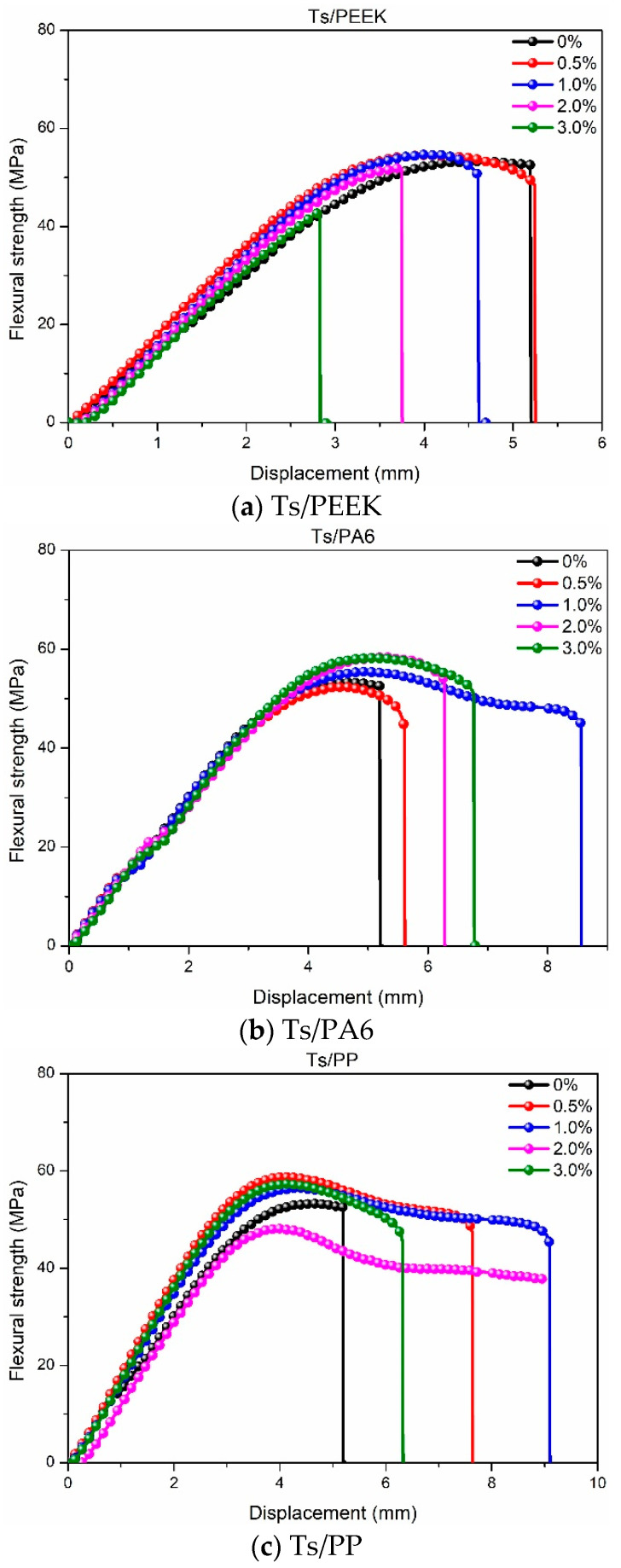
Effects of thermoplastic resin fillers on the flexural strength-displacement curves of Ts resin of (**a**) Ts/PEEK, (**b**) Ts/PA6 and (**c**) Ts/PP.

**Figure 8:**
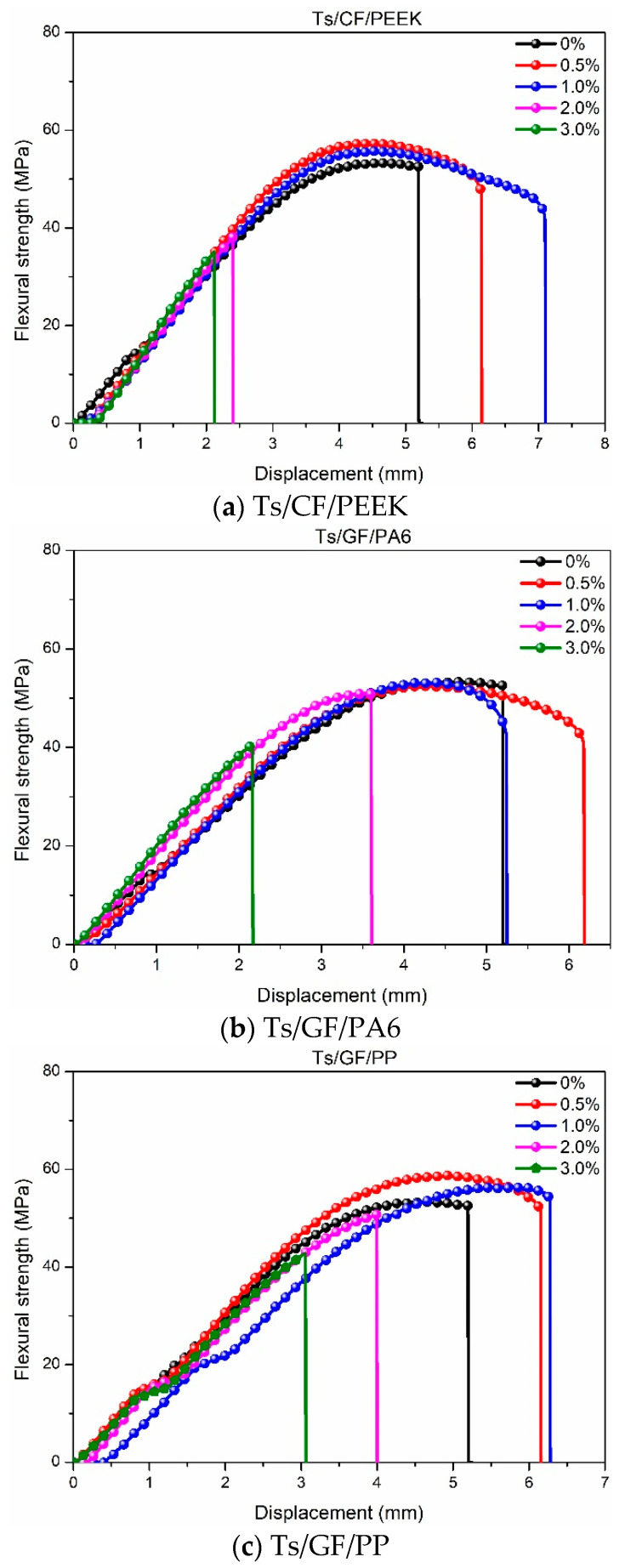
Effects of thermoplastic composite fillers on the flexural strength-displacement curves of Ts resin of (**a**) Ts/CF/PEEK, (**b**) Ts/GF/PA6 and (**c**) Ts/GF/PP.

After analyzing the tensile properties, the effects of thermoplastic fillers on the flexural properties of Ts resin were further investigated as shown in Figure 6, Figure 7 and Figure 8. As shown in Figure 6, It can be found that the addition of thermoplastic resin and composite fillers can both improve the flexural strength of Ts resin. Meanwhile, the improvement ratio decreased with the increase of filler mass ratio whether for resin filler or composite filler. This was attributed to the thermoplastic resin filler absorbed the energy generated by flexural load due to the high toughness and deformation ability. Excessive mass ratio led to the agglomeration effect of thermoplastic fillers in Ts resin, resulting in stress concentration and decrease of flexural strength. In addition, it can be found that PA6 and PEEK had superior enhancement effects to Ts resin compared to PP, which brough about the higher improvement of flexural strength.

Figure 7 and Figure 8 show the effects of thermoplastic resin and composite fillers on the flexural strength-displacement curves of Ts resin. As shown in Figure 7, the strength-displacement curves obviously moved to the right and up after adding PA6 and PP fillers, indicating the acquisition of higher strength and greater toughness of Ts resin. In contrast, the above enhancement and toughening effects were not obvious for the filler of PEEK. As shown in Figure 8, better reinforcement and toughening effects for Ts resin were found for the three composite fillers. Higher ultimate displacement was achieved when Ts resin had the flexural fracture, which can also provide an early warning signal by improving the toughness of Ts resin. It can be further predicted that after adding thermoplastic resin and composite fillers for the suitable mass ratio, the fatigue resistances of Ts resin will be further improved. In addition, the critical stress intensity factor (K_IC_) can be evaluated based on ASTM D5045-14 standard. The obvious improvement of the fracture toughness relative to the neat epoxy resin was observed, similar to the reports [39,48], which verified the addition of thermoplastic filler can significantly improve the fracture toughness of epoxy resin. However, when the filler content exceeded the optimal content, a decrease in fracture toughness was observed due to the agglomeration effect [47].

The increase percentage of flexural strength for Ts resin after adding the thermoplastic fillers was obtained based on Figure 6. For the resin filler, it can be found that the maximum increase percentages of flexural strength for Ts were 4.99% (PEEK), 12.55% (PA6) and 13.32% (PP), respectively. Similarly, the maximum flexural strength was increased by 15% through adding 20 phr polyetherketone cardo into epoxy resin [48]. For the composite filler, the increase percentages were 9.45% (CF/PEEK), 6.66% (GF/PA6) and 6.45% (GF/PP), respectively. To sum up, the flexural strength of Ts resin can be greatly improved when the mass ratio of thermoplastic resin and composite fillers were controlled at about 0.5~1%, which showed the similar variation trends with the tensile strength. Furthermore, the considerable flexural reinforcement and toughening effects were achieved for Ts resin.

#### 3.1.3. In-Plane Shear Strength

**Figure 9:**
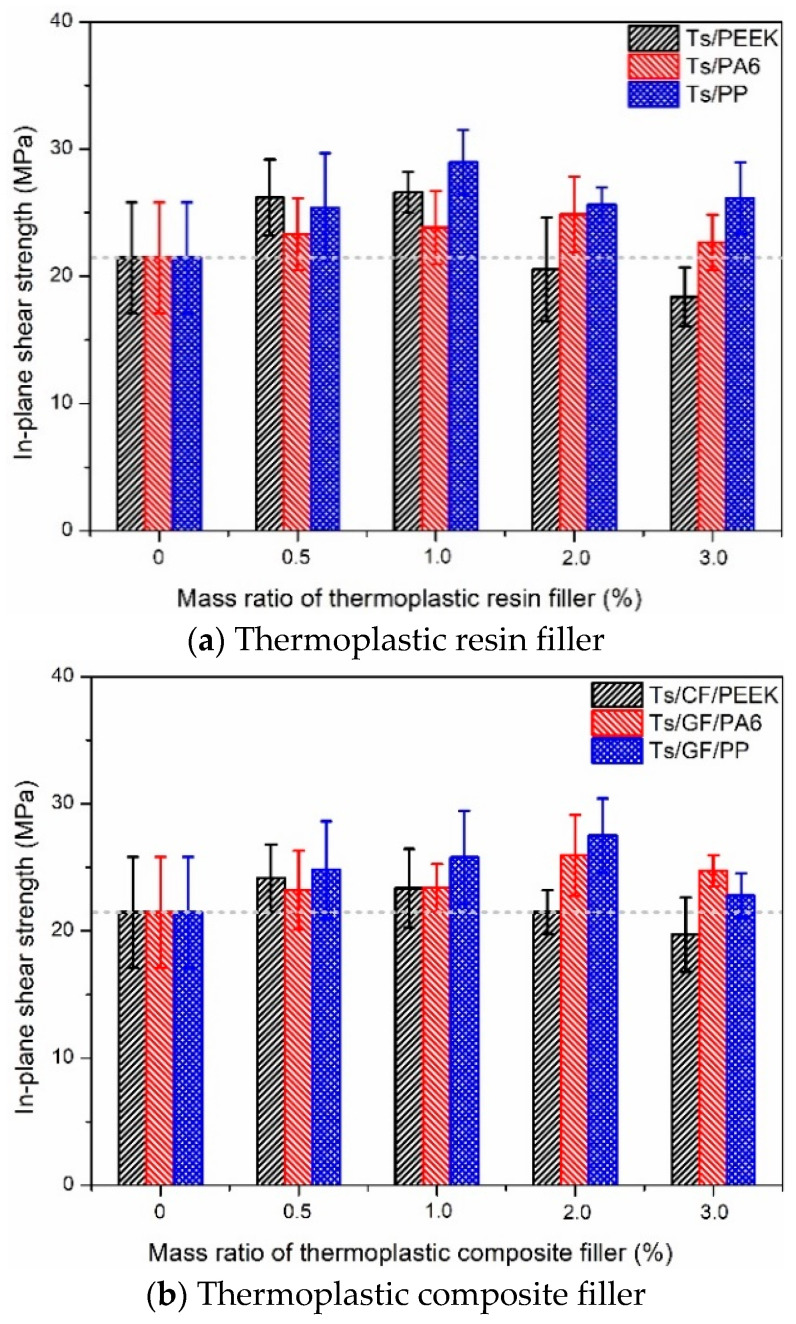
Effects of thermoplastic resin and composite fillers on the in-plane shear strength of Ts resin of (**a**) thermoplastic resin filler and (**b**) thermoplastic composite filler.

Figure 9 shows the effects of thermoplastic resin and composite fillers on the in-plane shear strength of Ts resin. Adding the thermoplastic resin and composite fillers can both improve the in-plane shear strength of Ts. Compared to PEEK filler, PA6 and PP had the more superior reinforcing effect for in-plane shear strength, which brough about the obvious improvement for all mass ratio. Furthermore, the increase percentages of in-plane shear strength for Ts resin were summarized. It can be found that the maximum increase percentages of in-plane shear strength were up to 20~30% and concentrated on the low mass ratio of 0.5~1.0%. In addition, PP and GF/PP fillers were more suitable to achieve the higher in-plane shear strength. The improvement mechanism of in-plane shear strength was attributed to the reinforced interface among the Ts, thermoplastic resin and carbon/glass fiber owing to the intermolecular and mechanical interactions.

### 3.2. Thermal Properties Analysis

#### 3.2.1. Dynamic Mechanical Analysis

**Figure 10:**
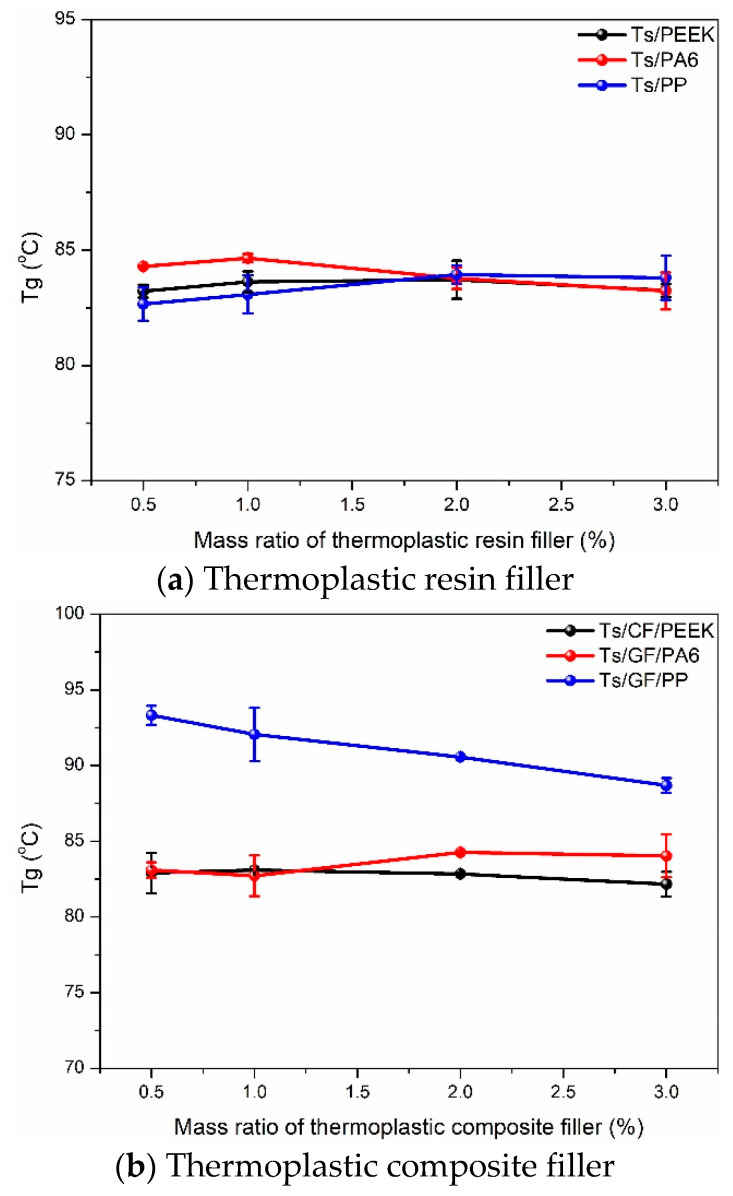
Effects of thermoplastic resin and composite fillers on Tg of Ts resin of (**a**) thermoplastic resin filler and (**b**) thermoplastic composite filler.

Dynamic mechanical analysis can characterize the thermodynamic properties of resin matrix composites, such as glass transition temperature, storage modulus, etc [49]. After analyzing the mechanical properties, the effects of thermoplastic resin and composite fillers on glass transition temperature (Tg) of Ts resin were obtained and shown in Figure 10. Before adding the thermoplastic fillers, Tg of Ts resin was 89.85 °C. After adding the thermoplastic resin, it can be found that Tg had a descent within 5 °C. Meanwhile, the mass ratio of filler had no obvious effect on the variations of Tg. The reason of Tg decrease was attributed to the addition of thermoplastic resin can reduce the curing degree of the epoxy and improved the flexibility of Ts resin due to the linear molecular structure of thermoplastic resin. Similar condition was also reported in the work [39]. For the filler of thermoplastic composite, there were two trends of Tg for three kinds of fillers. Adding the GF/PP filler brought about the increase of Tg and higher mass ratio of filler led to the decrease of Tg. In contrast, the effects of CF/PEEK and GF/PA6 fillers on Tg were similar to PEEK and PA6 fillers. The increase of Tg for Ts/GF/PP composite may be the superior interface bonding between fibers and resins [47], which was consistent with the significant increase of in-plane shear strength.

**Figure 11:**
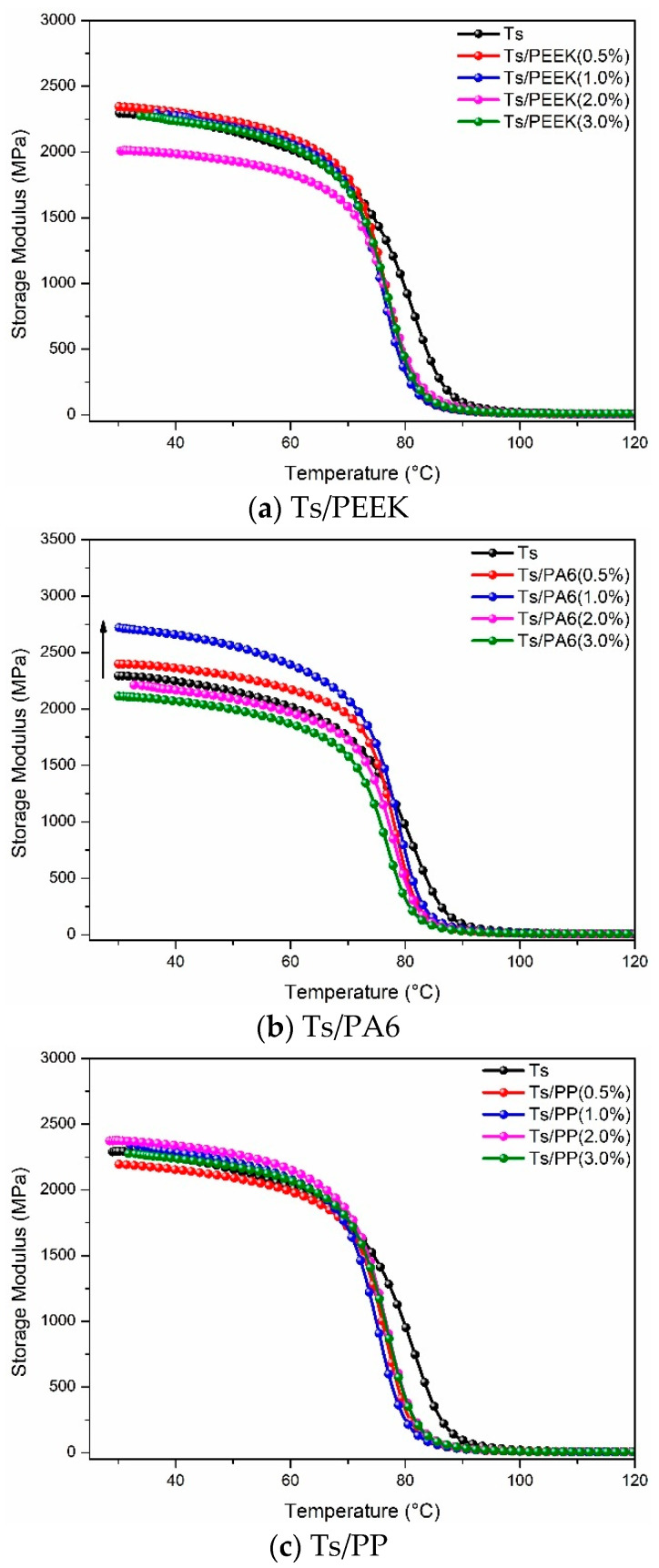
Effects of thermoplastic resin fillers on storage modulus of Ts resin of (**a**) Ts/PEEK, (**b**) Ts/PA6 and (**c**) Ts/PP.

**Figure 12:**
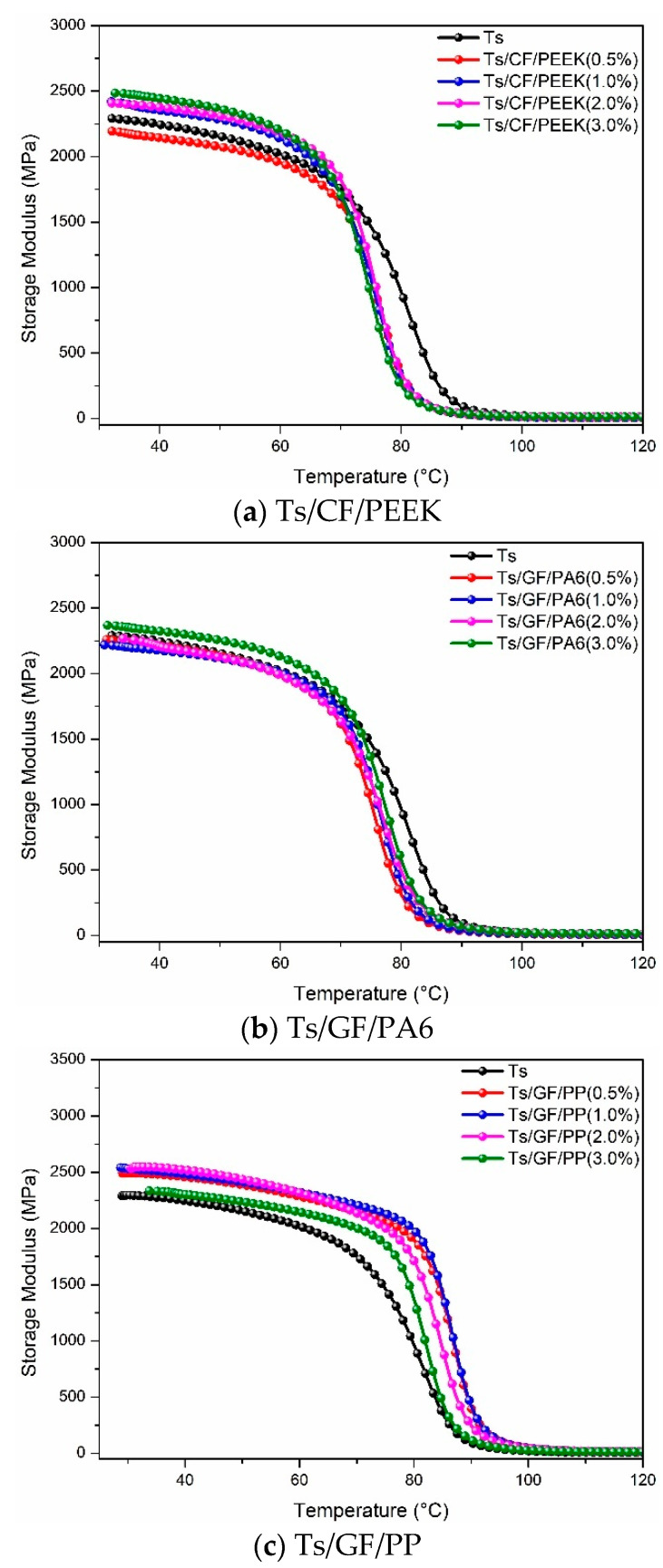
Effects of thermoplastic composite fillers on storage modulus of Ts resin of (**a**) Ts/CF/PEEK, (**b**) Ts/GF/PA6 and (**c**) Ts/GF/PP.

Figure 11 and Figure 12 show the effects of thermoplastic resin and composite fillers on storage modulus of Ts resin. For the thermoplastic resin filler (Figure 11), it can be found that adding PA6 can improve the storage modulus of Ts at a lower mass ratio, similar to the effect of polyimide [44]. By contrast, the effects of PEEK and PP on storage modulus of Ts can be ignored. In addition, within the main decrease area of storage modulus (70–90 °C), the storage modulus curves of control were always higher than that with thermoplastic resin filler, indicating the weakening of thermal properties owing to the decrease of curing degree of the epoxy [39,47]. As shown in Figure 12, the addition of CF/PEEK and GF/PA6 did not bring about the obvious increase of storage modulus. However, the significant improvement was observed for the filler of GF/PP whether for the initial storage modulus or decrease rate. Therefore, the overall analysis based on Tg and storage modulus showed that the thermoplastic composite filler of GF/PP was more conducive to improving the thermal properties of Ts resin.

#### 3.2.2. Thermogravimetric Analysis

**Figure 13:**
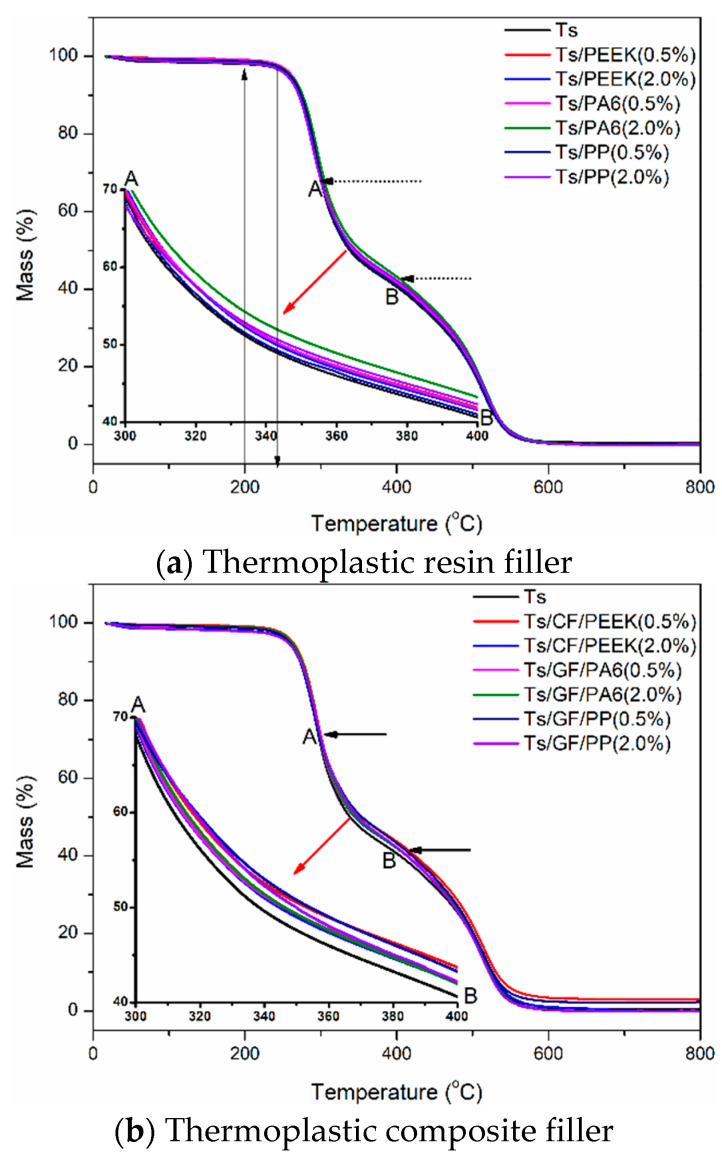
Effects of thermoplastic resin and composite fillers on weight loss of Ts resin at elevated temperatures of (**a**) thermoplastic resin filler and (**b**) thermoplastic composite filler. It was noted that AB denoted the typical decomposition zone.

Thermogravimetric analysis of Ts resin with or without the fillers were conducted and the weight variations at elevated temperatures were obtained as shown in Figure 13 and Table 2. It is worth mentioning that two kinds of addition ratios (0.5% and 2.0%) were used to evaluate the effect of thermoplastic filler on the thermal resistance of epoxy resin before and after the agglomeration effect. A two-stage weight loss was detected including the initial decomposition of the epoxy resin and subsequent the inherent high heat resistance properties of thermoplastic fillers [48]. As shown, the weight decomposition temperature zone of Ts and Ts/filler composites was approximately 200~600 °C, including two decomposition trends. Furthermore, the typical decomposition zone (AB) was magnified locally as shown in Figure 13. For the thermoplastic resin filler, it can improve the thermal decomposition capacity of Ts resin, leading to the higher residual weight. The remarkable increase of thermal resistance can be observed for the thermoplastic composite filler, especially for GF/PP, which was consistent with the dynamic mechanical analysis. A verification of the similar improvement in thermal resistance through adding polysulfone and polyimide into epoxy was also found in the research work of Zhang et al. [44].

Table 2 shows the quantitative effects of thermoplastic resin and composite fillers on the thermal properties of Ts resin. The temperature range of 250~500 °C was selected to evaluate the residual weight of composite at elevated temperature. It can be concluded that the increase percentage of residual weight at elevated temperature was up to 3%, indicating the obvious improvement of thermal resistance. In addition, the thermoplastic composite fillers had better enhancement effect on the thermal properties of Ts resin compared to the thermoplastic resin fillers. Furthermore, PP resin was more suitable as the fillers to improve the weight decomposition capacity of Ts resin at elevated temperature compared to PEEK and PA6.

### 3.3. Mechanism Analysis

#### 3.3.1. Functional Group Analysis

**Figure 14:**
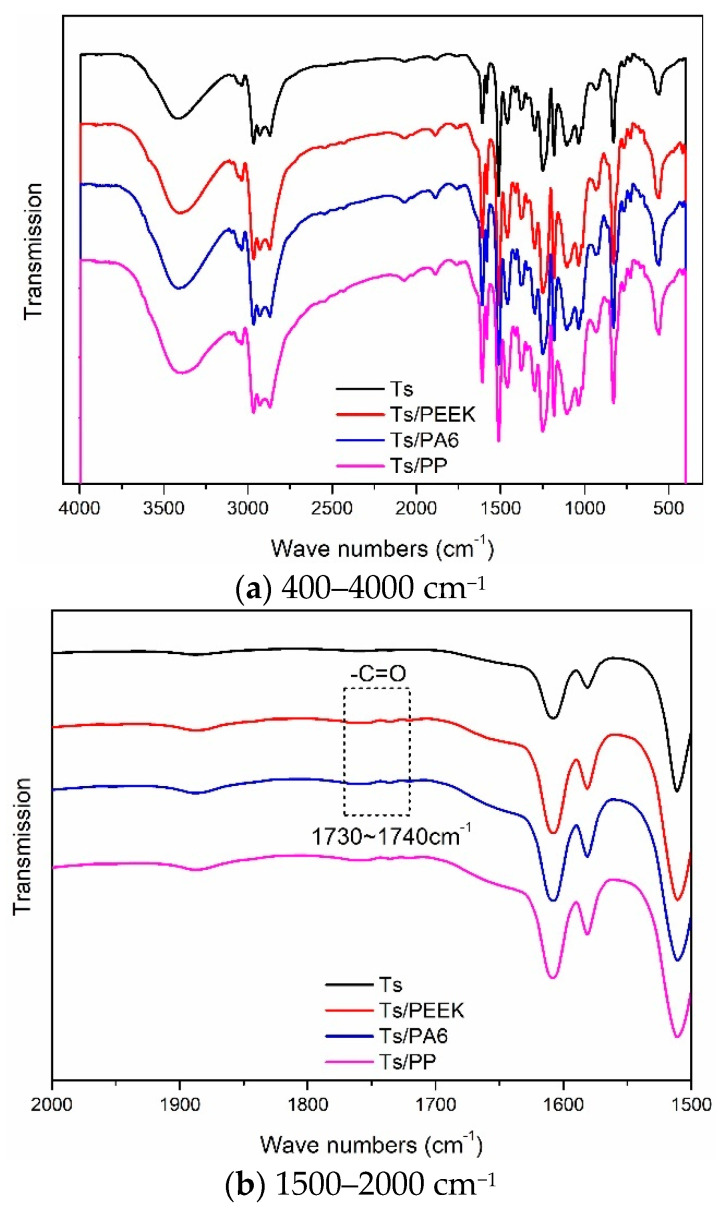
Effects of thermoplastic resin fillers on infrared transmission of Ts resin of (**a**) 400–4000 cm^−1^ and (**b**) 1500–2000 cm^−^^1^.

**Figure 15:**
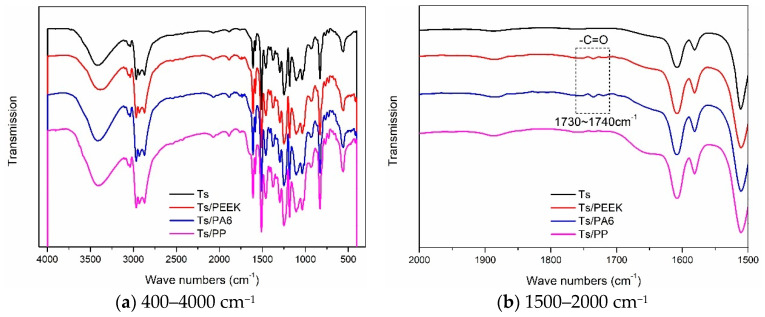
Effects of thermoplastic composite fillers on infrared transmission of Ts resin of (**a**) 400–4000 cm^−1^ and (**b**) 1500–2000 cm^−1^.

**Figure 16:**
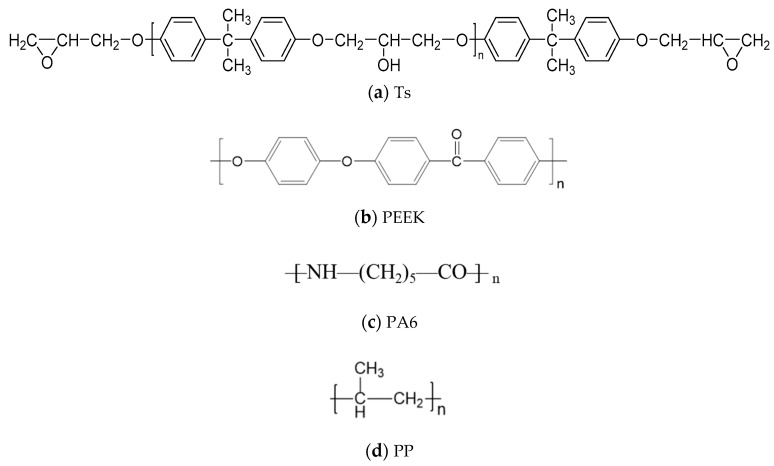
Molecular structure of Ts resin and three kinds of thermoplastic resin fillers of (**a**) Ts, (**b**) PEEK, (**c**) PA6 and (**d**) PP.

The functional groups of Ts and Ts/fillers were obtained through the infrared spectrum analysis and the effects of thermoplastic resin and composite fillers on infrared transmission of Ts resin were shown in Figure 14 and Figure 15. Furthermore, the molecular structure of Ts resin and three kinds of thermoplastic resin fillers were shown in Figure 16. As shown, it can be found that a new weak peak marked as –C=O appeared at the wave numbers of 1730–1740 cm^−1^ after adding the thermoplastic resin (PEEK and PA6) and their composite fillers. This was because there were –C=O functional groups in the molecular structure of PEEK and PA6, which has also proved that the above two thermoplastic resin fillers can be evenly dispersed in Ts resin. It should be noted that due to the low content of thermoplastic fillers, the above new peak was relatively weak. In contrast, the additions of PP resin and its composite filler did not bring about the above new peak owing to its molecular structure.

In the present paper, the amine curing agent (–NH_2_) was used as another component of Ts resin. Therefore, the prepared Ts resin contained N–H bond. Furthermore, the stretching vibration of the N–H bond was detected in the range of 3300–3500 cm^−1^ as shown in Figure 14 and Figure 15 whether the thermoplastic resin and composite fillers were added or not. In addition, the benzene ring contained in PEEK, C–C, C–H bonds contained in PP and N–H, C–C, C–H bonds contained in PA6 all existed in Ts epoxy resin, so no new vibration peak appeared. In conclusion, the above analysis showed that the thermoplastic resin and composite fillers can be evenly dispersed in Ts resin to further improve the mechanical and thermal properties.

#### 3.3.2. Surface Morphology Analysis

**Figure 17:**
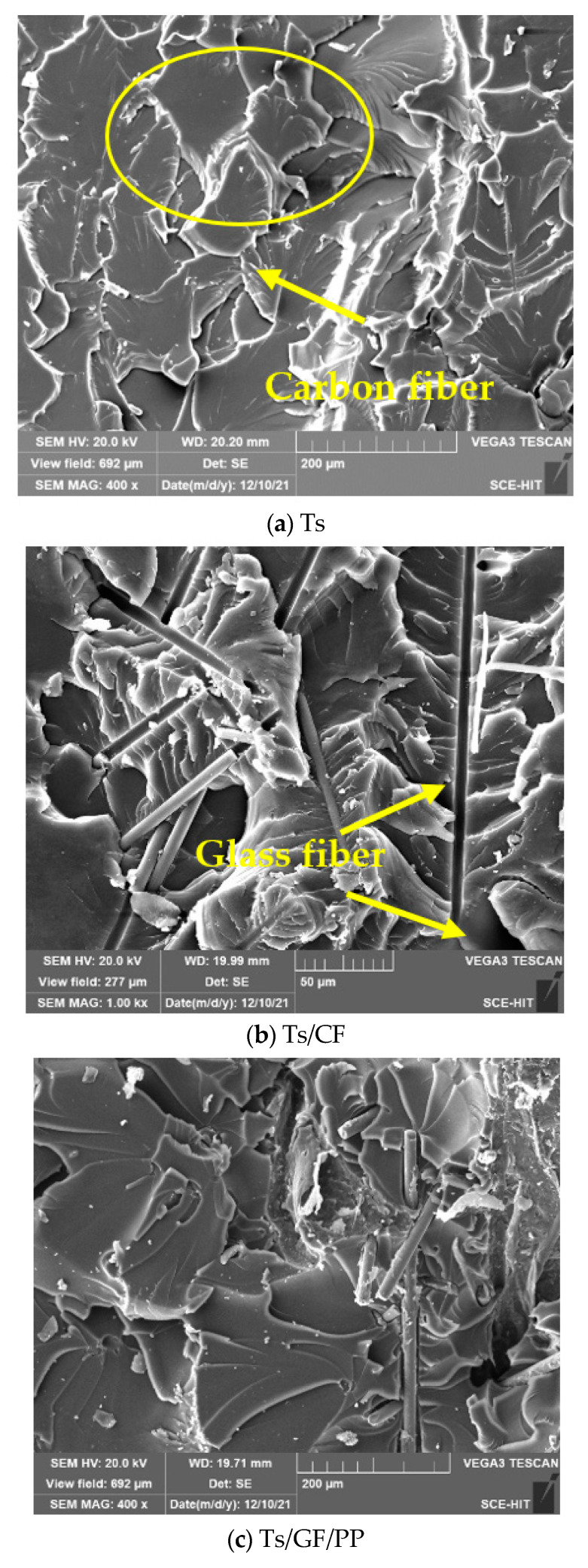

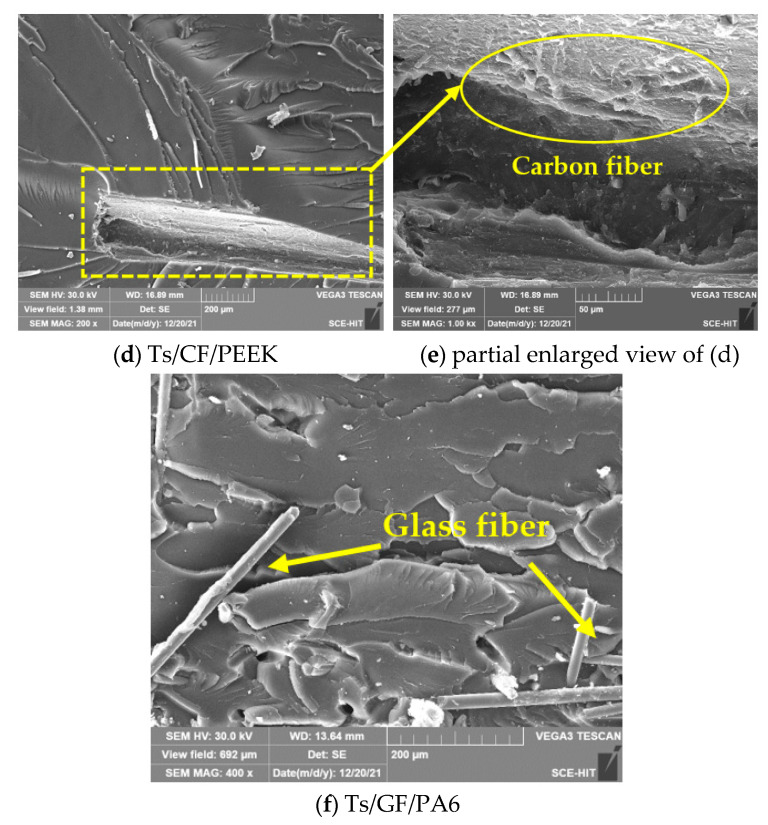
Effects of thermoplastic resin and composite fillers on tensile fracture morphology of Ts resin of (**a**) Ts, (**b**) Ts/CF, (**c**) Ts/GF/PP, (**d**) Ts/CF/PEEK, (**e**) partial enlarged view of (**d**) and (**f**) Ts/GF/PA6.

Figure 17 shows the effects of thermoplastic resin and composite fillers on tensile fracture morphology of Ts resin. It should be pointed out that three different magnifications were used to better observe the dispersion state of thermoplastic filler in epoxy resin owing to the different sizes of thermoplastic filler (such as the diameter of carbon fiber and glass fiber). It can be seen that the tensile fracture morphology of Ts resin (Figure 17a) presented on the typical fish scale shape. Some resin sheets showed the surface tearing, indicating the brittle failure characteristics. Figure 17b shows the carbon fibers were evenly dispersed in Ts resin. When the Ts resin had the tensile fracture, the carbon fiber was pulled out from the resin through absorbing part of the energy generated from the external load, which attributed to the improvement of mechanical properties. Figure 17c,d,f show the effects of three kinds of thermoplastic composites fillers on the tensile fracture morphology. It can be observed that carbon and glass fibers were dispersed in the Ts resin in the form of multi-directional orientation, which can provide a blocking path for crack propagation. In addition, some thermoplastic and Ts resins were observed to residue on the fiber surface, indicating the better interface bonding between the two resins and fibers.

#### 3.3.3. Mechanism Summary

**Figure 18:**
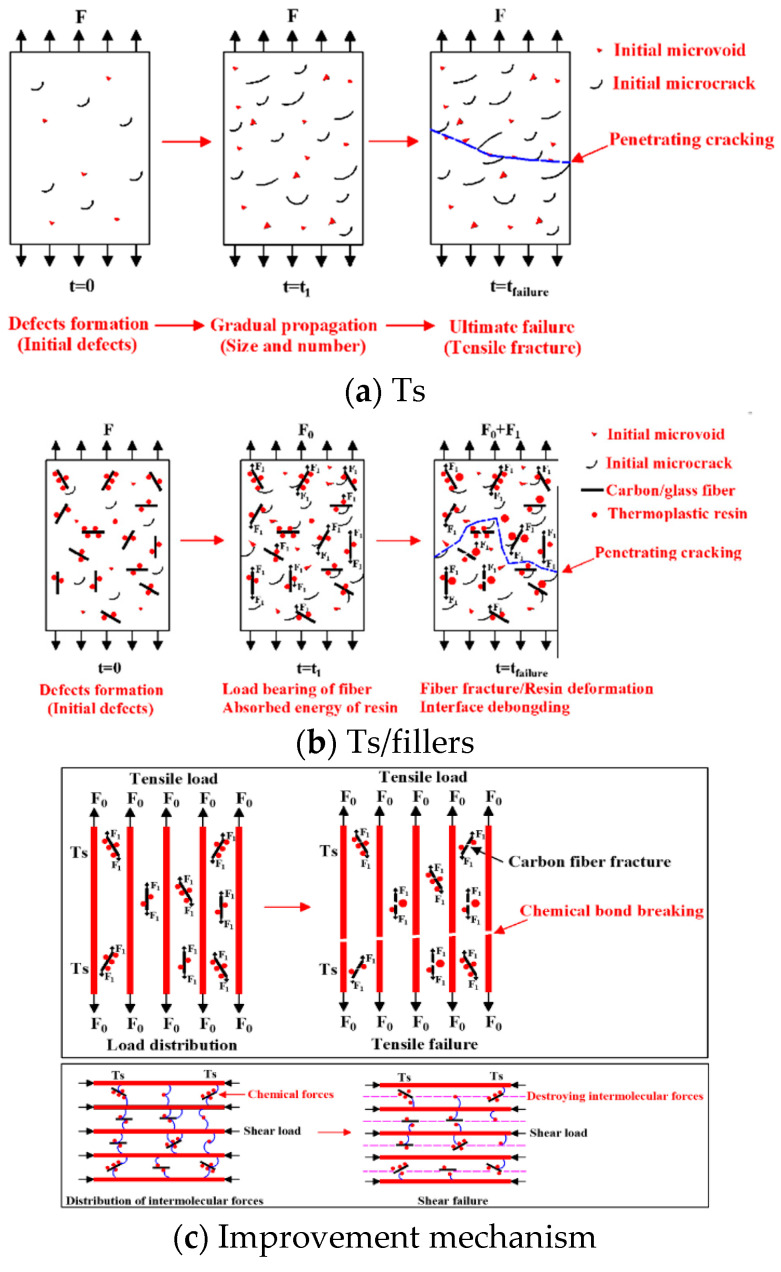
Effects of thermoplastic resin and composite fillers on failure mechanism of Ts resin of (**a**) Ts, (**b**) Ts/fillers and (**c**) improvement mechanism.

According to the above microstructure analysis, the improvement mechanism of mechanical properties through adding the thermoplastic resin and composite fillers into Ts resin was summarized in Figure 18. As shown in Figure 18a, it can be found that without the thermoplastic fillers, the tensile failure process of Ts resin was accompanied by the defect’s formation, gradual propagation and ultimate failure. The internal initial defects of Ts resin included the microvoids and microcracks, and the size and number gradually increased under the external load to form the penetrating cracking.

Figure 18b shows the effects of thermoplastic resin and composite fillers on failure mode of Ts resin. It can be found that the addition of thermoplastic fillers can prolong the cracking path and delay the failure process through the effects of fiber, resin and interface. For the fiber, its main function was to hinder the crack propagation and bear part of the external load. For thermoplastic resin, it can absorb the energy generated by crack propagation and produce higher elastic and plastic deformations. In addition, the interface bonding between the fiber and the two resins was improved through the intermolecular force, which can withstand part of external load. Therefore, when the mass proportion of thermoplastic filler was moderate, the enhancement effect of mechanical and thermal properties for Ts resin was prominent. However, if the mass proportion was too high, it would lead to the agglomeration and stress concentration of thermoplastic filler in Ts resin, leading to the decrease of mechanical and thermal properties.

Figure 18c shows the improvement mechanism of mechanical properties for Ts resin. The improvement mechanisms of flexural and shear performance were because the thermoplastic filler increased the intermolecular force of Ts resin, which brought about the increase of flexural and shear strength (Figure 6 and Figure 9). The improvement mechanism of tensile properties was carbon fiber and glass fiber born part of external load. Therefore, only the thermoplastic composite filler brought about the increase of tensile strength (Figure 4) instead of thermoplastic resin filler.

### 3.4. Proportioning Design of Optimal Thermoplastic Filler

According to the above analysis, the proportioning designs of optimal thermoplastic filler were obtained and shown in Table 3 and Table 4. It was worth mentioning that the selection of thermoplastic filler ratio was based on the improvement of mechanical and thermal properties of Ts resin. As shown in Table 3, it can be found that the optimal proportions of thermoplastic resin filler were 0.5~1.0% (PEEK), 1.0~2.0% (PA6) and 0.5~1.0% (PP) to obtain the desirable improvement of mechanical and thermal properties. In addition, it can be observed that the tensile strength of Ts resin did not increase for the thermoplastic resin filler owing to the absence of carbon fiber or glass fiber. Table 4 shows that adding three kinds of thermoplastic composite fillers can obviously improve the mechanical properties and thermal resistances, especially for GF/PP. Furthermore, the optimal mass ratios of three kinds of composite were determined to be 0.5~1.0%. For example, the filler of GF/PP mass ratio was 0.5%, the increase percentages of mechanical and thermal properties for Ts resin were 8.48% (tensile strength), 3.54% (flexural strength), 45.53% (in-plane shear strength), 3.87% (Tg) and 6.52% (residual weight), respectively. Higher mass ratio of thermoplastic composite fillers led to the agglomeration effect in Ts resin, bringing about the decrease of mechanical and thermal properties.

### 3.5. Application Prospect Analysis

Application prospect of adding thermoplastic resin and composite fillers to Ts resin mainly includes the recycling and reutilization of thermoplastic resin composites and high-performance application of Ts resin.

On the one hand, carbon fiber and glass fiber reinforced thermoplastic composites have been widely used in the fields of aerospace, automation, civil engineering, marine engineering and wind power and so on. If the above composites reach the service limits of materials and structures during the service, the recovery and utilization of composites are the key issues when considering the recycling utilization, environmental and resource protections. Therefore, it will be an effective way to recycle the thermoplastic resin composites by using them as fillers.

On the other hand, the brittleness and hydrophilicity of epoxy resin may lead to the fatigue damage and insufficient durability during the long-term service. How to improve the mechanical properties, toughness and long-term durability of epoxy resin is one of the effective means to prolong the service life of materials and structures. Through the research of this paper, it can be found that adding thermoplastic resin and composite fillers to epoxy resin can significantly improve the mechanical properties, toughness and thermal properties of epoxy resin (Ts). Furthermore, the hydrophobicity of thermoplastic resin filler can also improve the long-term durability of epoxy resin. Therefore, after the epoxy are reinforced and toughened, it can be used in the resin matrix, interface adhesive and anti-corrosion coating for the applications of repairing and reinforcement of civil structures.

## 4. Conclusions

In the present paper, three kinds of thermoplastic resins and composites were adopted as the fillers to reinforce and toughen the epoxy resin (Ts). Through using the homogenization machine, the above fillers were evenly dispersed in Ts resin to obtain the new composites. Mechanical, thermal properties and microscopic tests were carried out to evaluate the effect of filler on Ts performances. Furthermore, the improvement mechanism of mechanical and thermal properties for Ts resin was revealed. The design of optimal thermoplastic filler was proposed based on the performance improvement. Finally, the application prospect of adding thermoplastic resin and composite fillers to Ts resin was analyzed. The following conclusions can be drawn:

(1) Adding thermoplastic resin and composite fillers at the low mass ratio of 0.5~1.0% brought about the maximum improvement of the tensile strength (7~15%), flexural strength (7~15%) and in-plane shear strength (20~30%) of Ts resin. With the increase of filler content to 2.0~3.0%, Ts resin became weakened and brittle owing to the agglomeration effect.
(2) After adding the thermoplastic resin, Tg had a descent within 5 °C. This was attributed to the addition of thermoplastic resin improved the flexibility of Ts resin due to the linear molecular structure of thermoplastic resin. Adding the GF/PP filler brought about the increase (~4 °C) of Tg and weight decomposition capacity of Ts resin at elevated temperature compared to CF/PEEK and GF/PA6.
(3) Microstructure analysis showed that the thermoplastic resin and composite fillers can be evenly dispersed in Ts resin. The addition of thermoplastic fillers can prolong the cracking path and delay the failure process through the load bearing of fiber, energy absorption of thermoplastic resin and superior interface bonding.
(4) The optimal proportions of thermoplastic resin filler were 0.5~1.0% (PEEK), 1.0~2.0% (PA6) and 0.5~1.0% (PP) to obtain the desirable improvement of mechanical and thermal properties. For example, the maximum improvement of mechanical properties was up to 45.53% (in-plane shear strength). In contrast, the optimal mass ratios of three kinds of composite were determined to be 0.5~1.0%. After the improvement, Ts resin can be used as the resin matrix, interface adhesive and anti-corrosion coating.

## Figures and Tables

**Table 1:** Test conditions of adding thermoplastic resin and its composite to Ts resin.

Filler Type	Mass Ratio (%)	Mechanical Tests	Thermal Properties Tests
PEEK CF/PEEK	0.5	Tensile test Flexural test In-plane test	Dynamic mechanical analysis Thermogravimetric analysis
	1.0		
	2.0		
	3.0		
PA6 GF/PA6	0.5	Tensile test Flexural test In-plane test	Dynamic mechanical analysis Thermogravimetric analysis
	1.0		
	2.0		
	3.0		
PP GF/PP	0.5	Tensile test Flexural test In-plane test	Dynamic mechanical analysis Thermogravimetric analysis
	1.0		
	2.0		
	3.0		

**Table 2:** Effects of thermoplastic resin and composite fillers on the thermal properties of Ts resin.

Filler Type	Residual Weight of Composite at Elevated Temperature (%)					
	250 °C	300 °C	350 °C	400 °C	450 °C	500 °C
No	96.01	68.18	47.63	40.65	32.17	19.18
PEEK-0.5%	97.16	69.63	48.94	41.89	33.24	19.65
PEEK-2.0%	96.66	70.51	48.61	41.61	33.24	20.27
CF/PEEK-0.5%	97.32	70.92	50.57	43.73	35.34	22.18
CF/PEEK-2.0%	96.04	69.13	49.03	42.03	33.39	19.92
PA6-0.5%	97.18	70.05	48.92	41.76	33.06	19.73
PA6-2.0%	96.80	71.53	50.63	43.25	34.62	21.42
GF/PA6-0.5%	96.90	69.55	49.32	42.24	33.48	19.67
GF/PA6-2.0%	97.06	70.14	49.32	42.03	33.23	19.43
PP-0.5%	96.66	69.01	47.99	41.01	32.54	19.15
PP-2.0%	95.93	67.95	49.38	42.33	33.96	20.75
GF/PP-0.5%	96.59	69.53	50.82	43.30	34.41	20.53
GF/PP-2.0%	95.91	70.59	50.00	42.18	32.99	19.05

**Table 3:** Proportioning design of optimal resin filler and the improvement percentage of mechanical and thermal properties.

Filler Type and Mass Ratio	Increase Percentage of Tensile Strength (%)	Increase Percentage of Flexural Strength (%)	Increase Percentage of In-Plane Shear Strength (%)	Increase Percentage of Tg (%)	Increase Percentage of Residual Weight at Elevated Temperature (%)
PEEK-0.5	/	4.44	22.01	/	3.05
PEEK-1.0	/	4.99	23.85	/	/
PEEK-2.0	/	/	/	/	2.36
PEEK-3.0	/	/	/	/	/
PA6-0.5	/	3.02	8.59	/	2.73
PA6-1.0	/	9.49	10.95	/	/
PA6-2.0	/	12.55	15.81	/	6.40
PA6-3.0	/	11.29	5.57	/	/
PP-0.5	/	13.32	18.35	/	0.89
PP-1.0	/	10.12	34.99	/	/
PP-2.0	/	/	19.29	/	4.13
PP-3.0	/	9.87	21.81	/	/

Note, the residual weight was obtained at 400 °C.

**Table 4:** Proportioning design of optimal composite filler and the improvement percentage of mechanical and thermal properties.

Filler Type and Mass Ratio	Increase Percentage of Tensile Strength (%)	Increase Percentage of Flexural Strength (%)	Increase Percentage of In-Plane Shear Strength (%)	Increase Percentage of Tg (%)	Increase Percentage of Residual Weight at Elevated Temperature (%)
CF/PEEK-0.5	13.70	9.45	12.50	/	7.58
CF/PEEK-1.0	15.21	6.64	8.81	/	/
CF/PEEK-2.0	0.81	/	/	/	3.39
CF/PEEK-3.0	5.98	/	/	/	/
GF/PA6-0.5	11.45	3.22	8.07	/	3.91
GF/PA6-1.0	7.55	6.66	9.02	/	/
GF/PA6-2.0	/	/	20.86	/	3.39
GF/PA6-3.0	/	/	15.24	/	/
GF/PP-0.5	8.48	3.54	45.53	3.87	6.52
GF/PP-1.0	1.51	6.45	20.13	2.46	/
GF/PP-2.0	/	/	28.08	0.80	3.76
GF/PP-3.0	/	/	6.10	/	/

## Data Availability

Not applicable.
